# High glucose downregulates myocardin expression in rat glomerular mesangial cells via the ERK signaling pathway

**DOI:** 10.18632/oncotarget.20498

**Published:** 2017-08-24

**Authors:** Ming Li, Lijuan Xu, Guowei Feng, Yan Zhang, Xin Wang, Yuebing Wang

**Affiliations:** ^1^ School of Basic Medical Sciences, Hebei University, Baoding, China; ^2^ Department of Biochemistry, School of Medicine, Nankai University, Tianjin, China; ^3^ Department of Genitourinary Oncology, Tianjin Medical University Cancer Institute and Hospital, National Clinical Research Center for Cancer, Key Laboratory of Cancer Prevention and Therapy, Tianjin, China; ^4^ Tianjin's Clinical Research Center for Cancer, Tianjin, China

**Keywords:** extracellular signal-regulated kinase (ERK), glomerular mesangial cells, high glucose, myocardin, smooth muscle α-actin (SM α-actin)

## Abstract

Mesangial cells (MCs), which are vascular smooth muscle-derived cells, occupy the central position in the glomerulus. Diabetic nephropathy (DN) is one of the most common diabetes complications and is likely attributed to the loss of MC contractility. Myocardin stimulates downstream vascular smooth muscle genes and regulates the contractility of vascular smooth muscle cells. Therefore, we hypothesized that *myocardin* is expressed in MCs and that high glucose is involved in the regulation of myocardin and downstream contractile genes in the context of DN. Confocal microscopy revealed that *myocardin* is expressed in rat MCs. Western blot and RT-qPCR analyses showed that treatment with 30 mM D-glucose significantly downregulated the mRNA and protein levels of myocardin and downstream SM α-actin. As an isotonic contrast, 30 mM mannitol did not affect *myocardin* mRNA levels but did downregulate myocardin protein levels. Treatment with 30 mM mannitol also downregulated SM α-actin mRNA and protein levels. Conversely, as another isotonic contrast, 30 mM L-glucose also had no effect on myocardin and SM α-actin expression in MCs. The extracellular signal-regulated kinase (ERK) pathway was activated by treatment with 30 mM D-glucose or mannitol, while specific inhibitors of the ERK pathway (PD98059) compromised the downregulation of myocardin and SM α-actin triggered by high glucose or mannitol. Thus we revealed that *myocardin* is expressed in MCs and that high glucose downregulates myocardin expression and downstream contractile protein SM α-actin via the ERK pathway. Our results suggest a novel mechanism for high glucose inhibition of MC contraction, which contributes to DN pathogenesis.

## INTRODUCTION

Diabetic nephropathy (DN) is a “microvascular’’ morbid complication associated with diabetes mellitus (DM) and is the most common cause of end-stage renal disease [1, 2]. Glomerular hyperfiltration is observed in patients with type 1 and type 2 DM and commonly occurs in the early stages of DN. Glomerular hyperfiltration has been recognized as a risk factor for subsequent increases in albuminuria and future progressive DN [3]. Until now, there has been no effective therapy for end-stage renal disease except kidney transplantation; therefore, early stage intervention plays a vital role in its treatment in DN patients.

Mesangial cells (MCs) are smooth muscle-like pericytes that abut and surround the filtration capillaries within the glomerulus [4, 5]. MCs are normally contractile cells that are strategically oriented among the glomerular capillary network to regulate the filtration surface area. A previous study reported that glomerular hyperfiltration will occur if the mesangial cells cannot isometrically and isotonically contract to counter intra-glomerular pressure [5], suggesting a role for mesangial cell contractility in the pathogenesis of DN. However, details regarding adaptor proteins and signaling pathways are still largely unknown.

Myocardin, the most important transcriptional coactivator of serum response factor (SRF) [6], plays a critical role in the development of vascular smooth muscle cells (VSMCs) contractile phenotype. The binding of myocardin to SRF at CArG box-containing target genes can transcriptionally activate a variety of downstream contractile genes, such as *smooth muscle α-actin* (*SM α-actin*), *Sm22α*, and *Acta2* [7, 8]. Our study has shown that downregulation of myocardin and downstream contractile proteins will reduce the contractility of VSMCs, contributing to the pathogenesis of cardiovascular disease hypertension [9], which serves as a stimulus for us to examine the regulation of myocardin expression in glomerular MCs. It has been revealed that *myocardin* is expressed in glomerular podocytes [10]; moreover, the myocardin downstream contractile gene *SM α-actin* is expressed in MCs [11–13]. However, little is known about the expression of *myocardin* in renal MCs or its regulation mechanism.

The present study was designed to characterize the effects of high glucose on myocardin expression and intracellular signaling pathways in rat glomerular MCs. Our study aimed to establish a potential role for high glucose/myocardin in the pathogenesis of DN.

## RESULTS

### *Myocardin* is expressed in rat glomerular MCs

**Figure 1:**
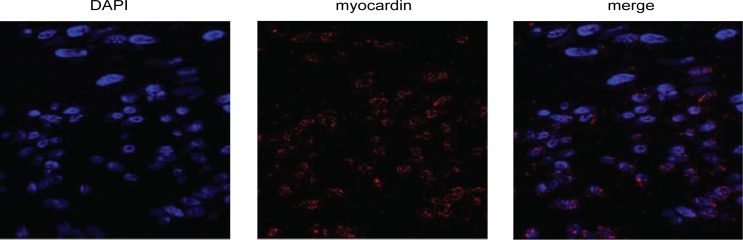
*Myocardin* is expressed in rat glomerular mesangial cells (MCs) Representative micrographs show immunostaining for myocardin (red) in rat glomerular MCs with the nuclei counterstained with DAPI (blue). The MCs were stained with myocardin antibody and Alexa Fluor 568-conjugated secondary antibody, followed by confocal microscopy as described in the Methods.

We first performed immunohistochemistry and confocal microscopy to visualize the myocardin protein in rat glomerular MCs. As shown in Figure 1, myocardin signals appeared inside the nuclei and overlapped with the DAPI nuclear staining.

**Figure 2:**
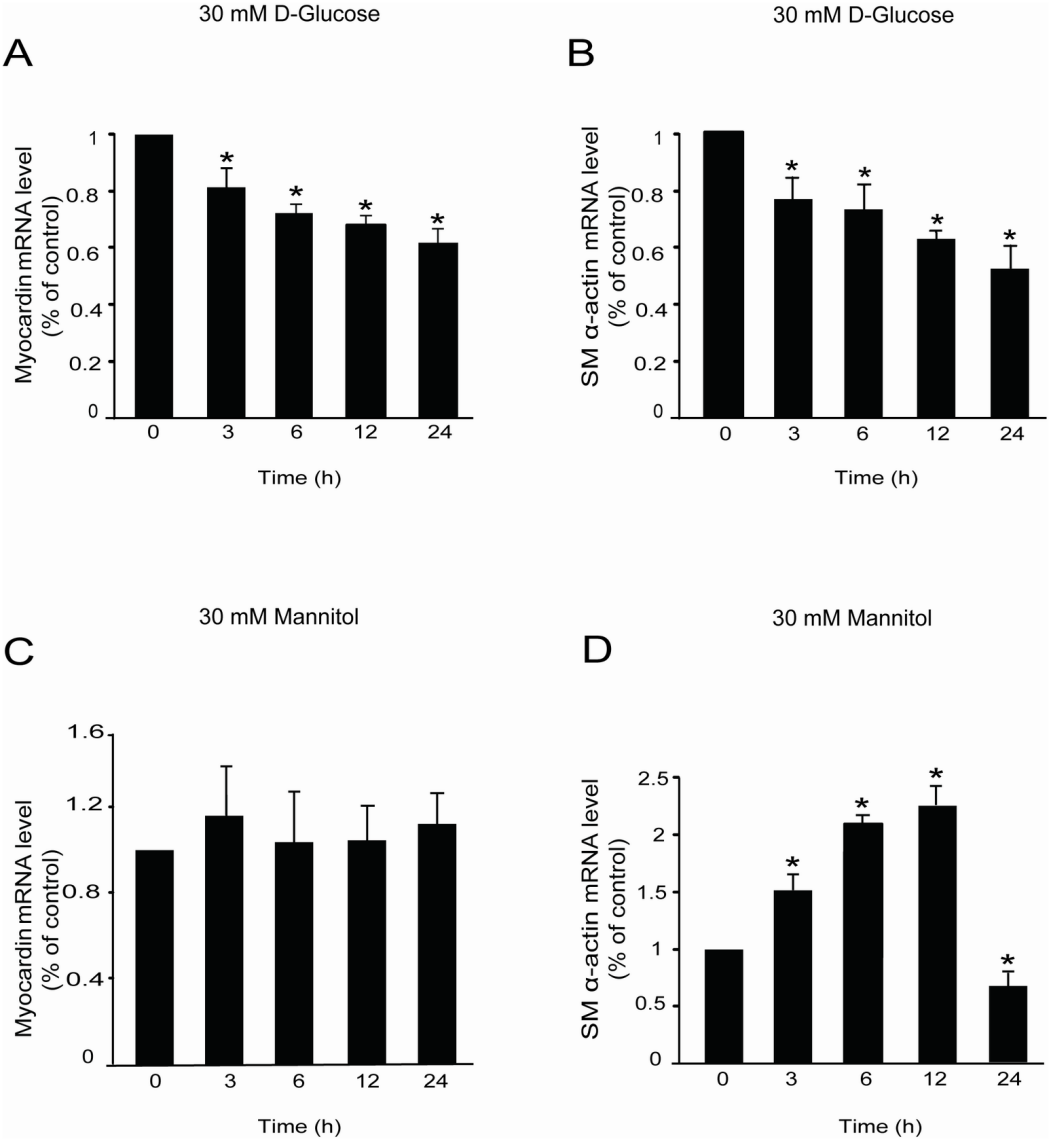
High glucose decreased *myocardin* and *SM α-actin* mRNA levels Rat MCs were rendered quiescent in DMEM low glucose medium containing 0 % fetal bovine serum for 24 h and then treated with or without 30 mM D-glucose or mannitol for the indicated times. RNA was extracted for RT-qPCR analyses. **(A)** RT-qPCR data showing *myocardin* mRNA levels in rat glomerular MCs with or without 30 mM D-glucose treatment for different times. **(B)** RT-qPCR data showing *SM α-actin* mRNA levels in rat glomerular MCs with or without 30 mM D-glucose treatment for different times. **(C)** RT-qPCR data showing *myocardin* mRNA levels in rat glomerular MCs with or without 30 mM mannitol treatment for different times. **(D)** RT-qPCR data showing *SM α-actin* mRNA levels in rat glomerular MCs with or without 30 mM mannitol treatment for different times. RT-qPCR results were normalized to *β-actin*. ^*^P < 0.05 vs. cells without any treatment. The results shown are representative of three experiments with different cell preparations.

**Figure 3:**
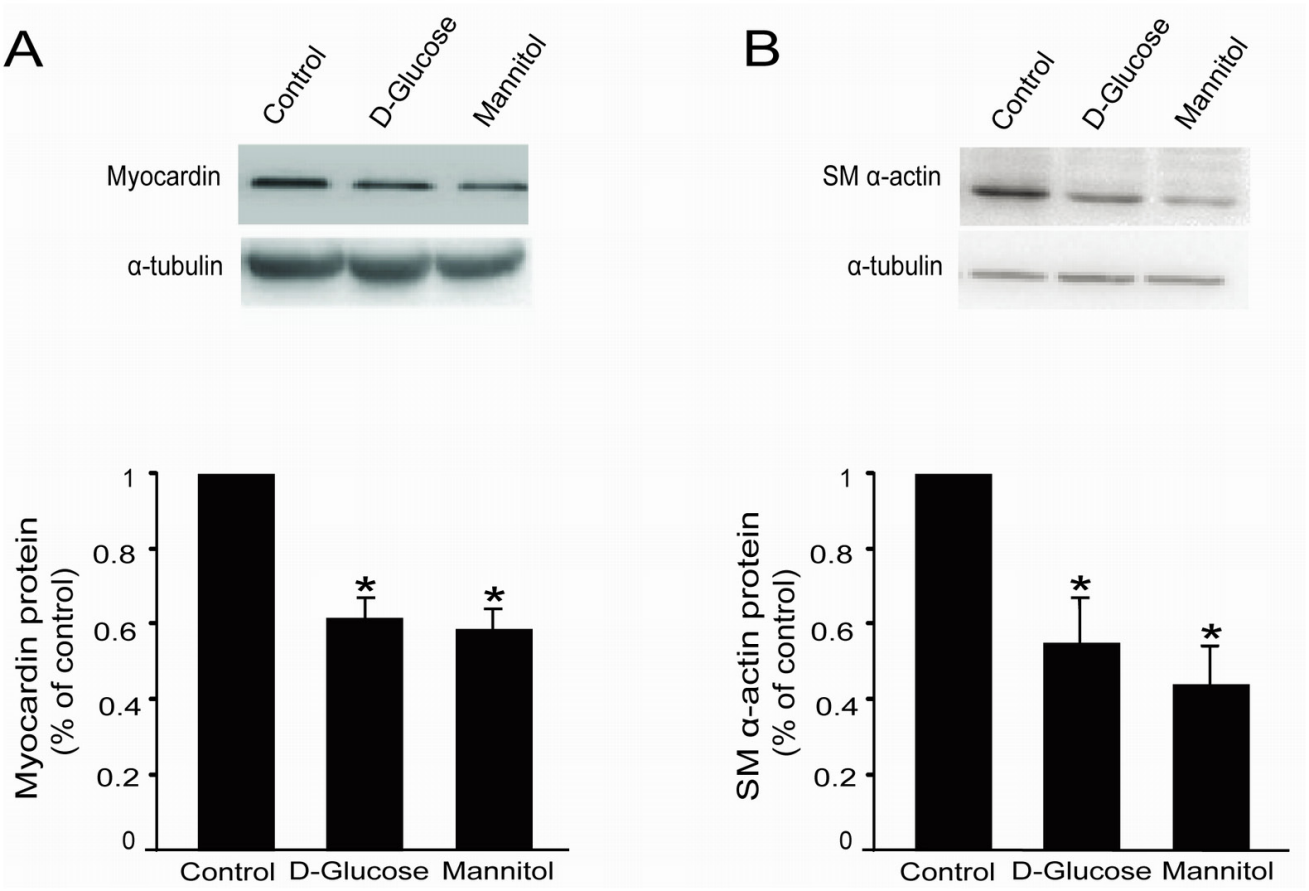
High glucose decreased myocardin and SM α-actin protein levels in rat glomerular MCs Rat MCs were rendered quiescent in low-glucose DMEM medium containing 0 % fetal bovine serum for 24 h, and then treated with or without 30 mM D-glucose or mannitol. Protein was extracted for Western blot analyses. **(A)** Cumulative Western blot results showing myocardin protein levels with or without 30 mM D-glucose or mannitol for 24 h. Insert: Representative Western blots of myocardin and α-tubulin. **(B)** Cumulative Western blot results showing SM α-actin protein levels in rat glomerular MCs with or without 30 mM D-glucose or mannitol for 24 h. Insert: Representative Western blots of SM α-actin and α-tubulin. Western blots results were normalized to α-tubulin. ^*^P < 0.05 vs. cells without any treatment. The results shown are representative of three experiments with different cell preparations.

To confirm this finding, we examined the mRNA and protein expression of myocardin in rat MCs. RT-qPCR analysis using specific primers showed that *myocardin* mRNA is expressed in rat glomerular MCs (Figure 2). Western blot analysis showed that myocardin protein is also expressed in MCs (Figure 3).

### High glucose decreases the mRNA levels of *myocardin* and *SM α-actin* in rat glomerular MCs

After confirming the expression of myocardin in rat glomerular MCs, the rat MCs cultured in DMEM low glucose medium were treated with 30 mM D-glucose or 30 mM mannitol (as an isotonic contrast) to investigate the mRNA expression of *myocardin* and the downstream contractile gene *SM α-actin*. RT-qPCR analysis showed that high glucose significantly downregulated the mRNA levels of both *myocardin* and *SM α-actin* in the rat glomerular MCs from 3 h to 24 h (Figure 2A & 2B). However, as an isotonic contrast, 30 mM mannitol did not decrease *myocardin* mRNA levels (Figure 2C) but did induce *SM α-actin* mRNA expression in a time-dependent manner before a decrease at 24 h (Figure 2D).

### High glucose decreases myocardin and SM α-actin protein levels in rat glomerular MCs

The rat glomerular MCs were treated with 30 mM D-glucose or mannitol for 24 h, followed by Western blot to examine myocardin and SM α-actin protein expression. Western blot results showed that 30 mM D-glucose significantly downregulated myocardin and downstream SM α-actin protein expression (Figure 3A & 3B). Similarly, 30 mM mannitol also downregulated myocardin and SM α-actin protein levels (Figure 3A & 3B).

### L-glucose did not affect myocardin and SM α-actin expression in rat glomerular MCs

**Figure 4:**
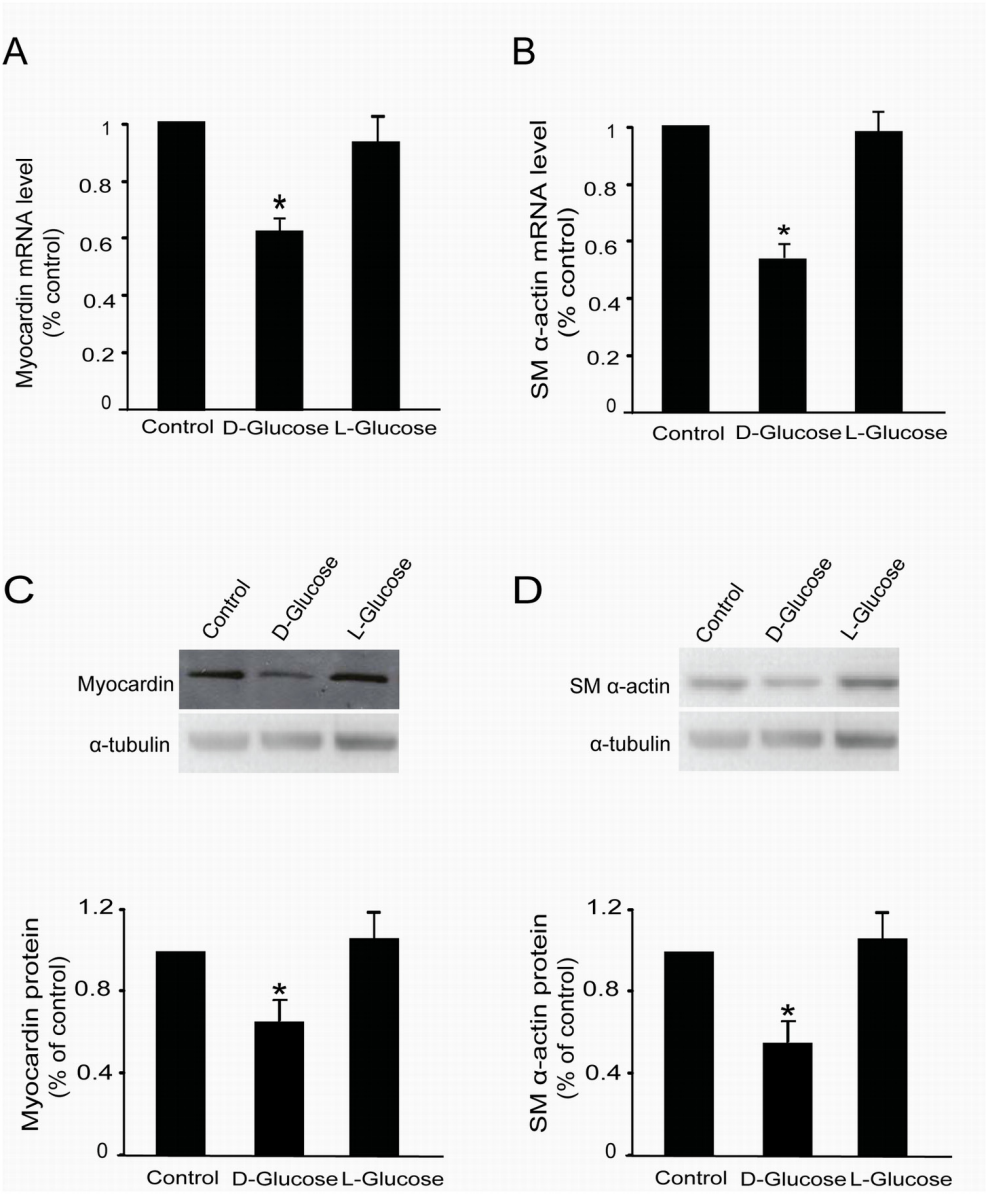
L-glucose did not regulate myocardin and SM α-actin expression Rat MCs were rendered quiescent in low–glucose DMEM medium containing 0 % fetal bovine serum for 24 h and then treated with or without 30 mM D-glucose or L-glucose. Total RNA and protein were extracted for RT-qPCR and Western blot analyses, respectively. **(A)** RT-qPCR data showing *myocardin* mRNA levels with or without 30 mM L-glucose treatment for 24 h. **(B)** RT-qPCR data showing *SM α-actin* mRNA levels with or without 30 mM L-glucose treatment for 24 h. **(C)** Cumulative Western blot results showing myocardin protein levels with or without 30 mM L-glucose treatment for 24 h. Insert: Representative Western blots of myocardin and α-tubulin. **(D)** Cumulative Western blot results showing SM α-actin protein levels with or without 30 mM L-glucose treatment for 24 h in rat glomerular MCs. Insert: Representative Western blots of SM α-actin and α-tubulin. RT-qPCR results were normalized to β-actin, and the Western blot results were normalized to α-tubulin. ^*^P < 0.05 vs. cells without any treatment. The results shown are representative of three experiments with different cell preparations.

Unexpectedly, it was observed that mannitol affected the expression of myocardin and SM α-actin in rat MCs; it did not function as an isotonic control. Therefore, we used L-glucose, an optical isomer of D-glucose, as another isotonic control to explore whether the effects of high glucose on myocardin and SM α-actin is caused by the osmotic potential. Rat glomerular MCs were treated with 30 mM D-glucose or L-glucose for 24 h; thereafter, total mRNA and proteins were extracted. RT-qPCR and Western blot were used to detect the mRNA and protein levels of myocardin and SM α-actin, respectively. Our results showed that L-glucose did not regulate myocardin or SM α-actin mRNA (Figure 4A & 4B) or protein (Figure 4C & 4D) levels in rat glomerular MCs.

### High glucose activates the ERK signaling pathway in rat glomerular MCs

**Figure 5:**
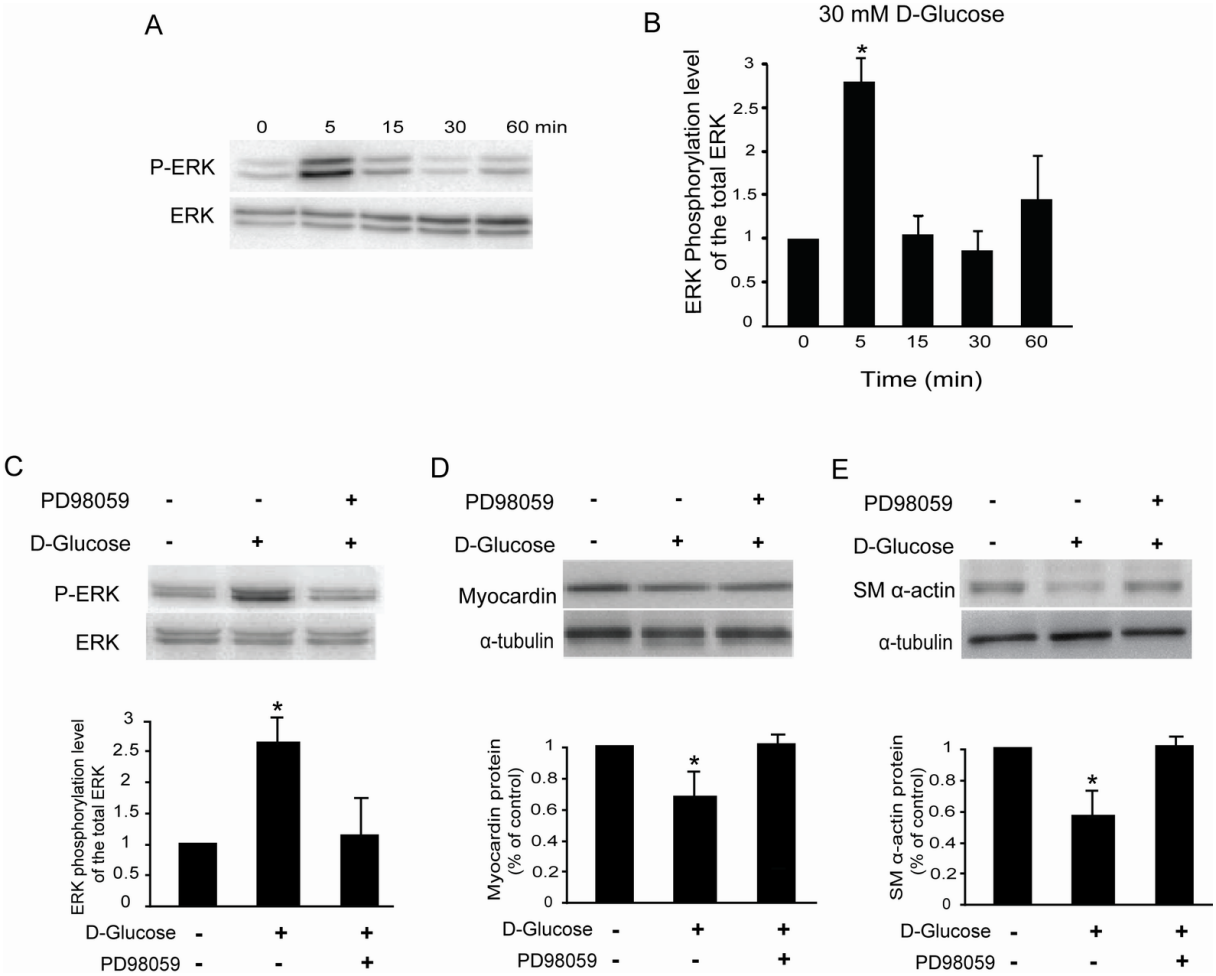
High glucose activates the ERK pathway in rat glomerular MCs **(A)** Representative Western blot results showing the phosphorylation of ERK1/ERK2 in rat MCs. **(B)** Cumulative Western blots showing the phosphorylation of ERK1/ERK2 in rat MCs treated with or without 30 mM D-glucose for different times. The y-axis represents the relative levels of phospho-ERK1/ERK2 normalized to total ERK1/ERK2 (^*^P < 0.05 vs. cells without any treatment). **(C)** Cumulative Western blots showing the phosphorylation of ERK1/ERK2 in rat MCs treated with or without D-glucose (30 mM) and PD98059 (10 μM). Inserts: Representative Western blot results showing the phosphorylation of ERK1/ERK2 in rat MCs. The y-axis represents the relative levels of phospho-ERK1/ERK2 normalized to total ERK1/ERK2 (^*^P < 0.05 vs. cells without any treatment). **(D)** Cumulative Western blot results showing the expression of myocardin in rat MCs treated with or without D-glucose (30 mM) and PD98059 (10 μM) (^*^P < 0.05 vs. cells without any treatment). Inserts: Representative Western blots of myocardin and α-tubulin. **(E)** Cumulative Western blot results showing the expression of SM α-actin in rat MCs treated with or without D-glucose (30 mM) and PD98059 (10 μM) (^*^P < 0.05 vs. cells without any treatment). Inserts: Representative Western blots of SM α-actin and α-tubulin.

It is reported that the extracellular signal-regulated kinase (ERK) signaling pathway is activated in hyperglycemia induced both DN and chronic renal failure [14], and we also examined the effects of high glucose on ERK1/ERK2 phosphorylation in rat glomerular MCs. As shown in Figure 5A & 5B, 30 mM D-glucose increased the ratio of phospho-ERK1/ERK2 and total ERK1/ERK2 in rat MCs as early as 5 min after treatment, and reached a peak at 5 min. Pretreatment with PD98059 (10 μM, 30 min), a specific MEK inhibitor, suppressed the ERK1/ERK2 phosphorylation induced by high glucose in rat MCs (Figure 5C).

Having shown that the ERK signaling pathway is activated by high glucose in rat MCs, we addressed whether ERK1/ERK2 phosphorylation is required for high glucose-inhibited myocardin and SM α-actin expression. Pretreatment of rat MCs with PD98059 (10 μM) compromised glucose-inhibited myocardin and SM α-actin protein levels (Figure 5D & 5E), as detected by Western blot. Consistently, 5 μM U0126, a mechanistically different inhibitor of ERK activation, also compromised the myocardin and SM α-actin protein levels downregulated by 30 mM D-glucose in rat MCs (data not shown). Therefore, we concluded that the ERK signaling pathway is involved in the downregulation of myocardin and downstream genes induced by high glucose in rat MCs.

### Mannitol activates the ERK pathway in rat glomerular MCs

**Figure 6:**
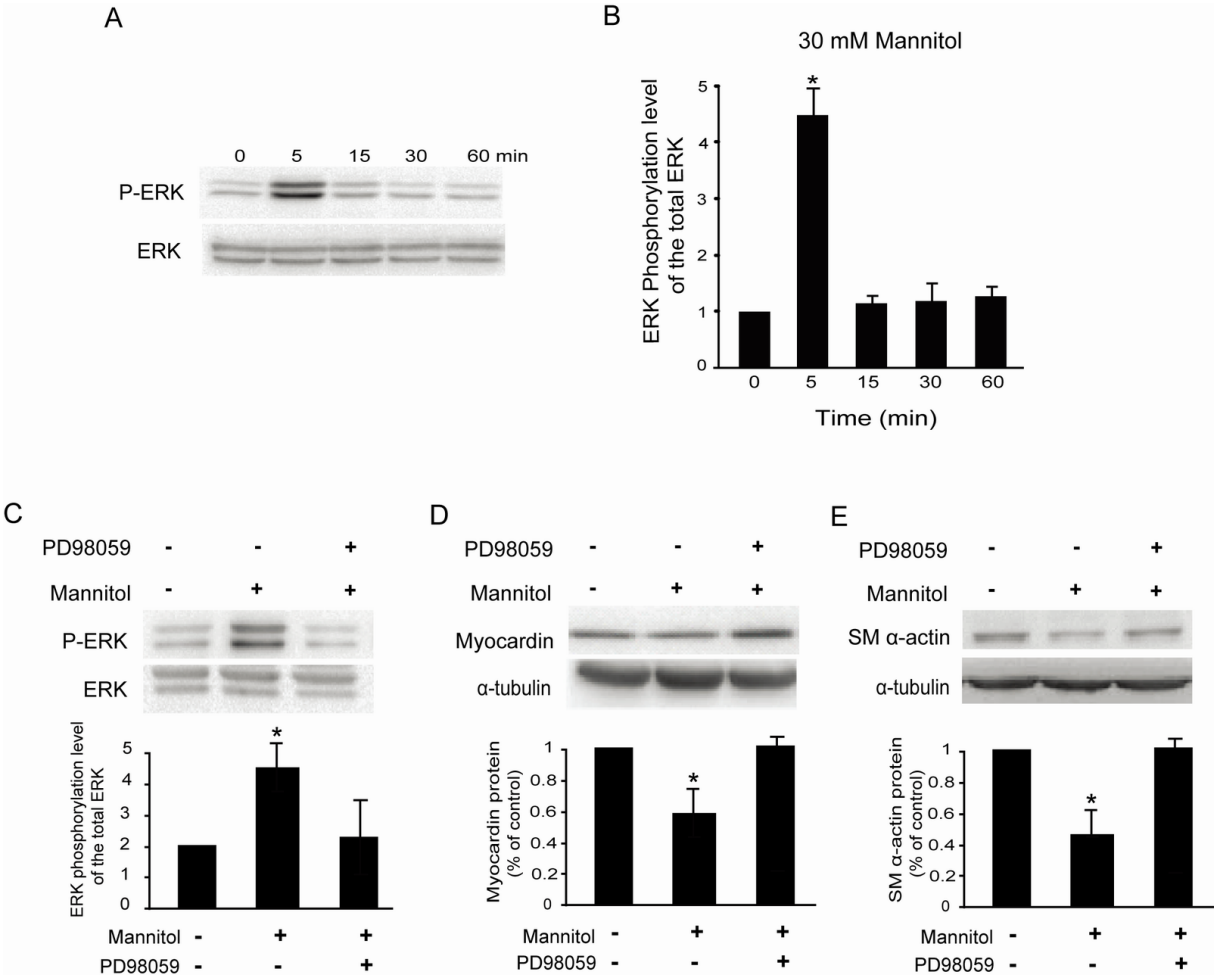
Mannitol activates the ERK pathway in rat glomerular MCs **(A)** Representative Western blot results showing the phosphorylation of ERK1/ERK2 in rat MCs. **(B)** Cumulative Western blots showing the phosphorylation of ERK1/ERK2 in rat MCs treated with or without 30 mM mannitol for different times. The y-axis represents the relative levels of phospho-ERK1/ERK2 normalized to total ERK1/ERK2 (^*^P < 0.05 vs. cells without any treatment). **(C)** Cumulative Western blots showing the phosphorylation of ERK1/ERK2 in rat MCs treated with or without mannitol (30 mM) and PD98059 (10 μM). Inserts: Representative Western blot results showing the phosphorylation of ERK1/ERK2 in rat MCs. The y-axis represents the relative levels of phospho-ERK1/ERK2 normalized to total ERK1/ERK2 (^*^P < 0.05 vs. cells without any treatment). **(D)** Cumulative Western blot results showing the expression of myocardin in rat MCs treated with or without mannitol (30 mM) and PD98059 (10 μM) (^*^P < 0.05 vs. cells without any treatment). Inserts: Representative Western blots of myocardin and α-tubulin. **(E)** Cumulative Western blot results showing the expression of SM α-actin in rat MCs treated with or without mannitol (30 mM) and PD98059 (10 μM) (^*^P < 0.05 vs. cells without any treatment). Inserts: Representative Western blots of SM α-actin and α-tubulin.

Because mannitol significantly downregulated the expression of myocardin, we examined the effects of mannitol on ERK1/ERK2 phosphorylation in rat glomerular MCs. As shown in Figure 6A & 6B, 30 mM mannitol increased the ratio of phospho-ERK1/ERK2 and total ERK1/ERK2 in rat MCs as early as 5 min after treatment and reached a peak at 5 min. Pretreatment with PD98059 (10 μM, 30 min), a specific MEK inhibitor, suppressed the ERK1/ERK2 phosphorylation induced by mannitol in rat MCs (Figure 6C).

We also examined whether ERK1/ERK2 phosphorylation is required for mannitol-inhibited myocardin and SM α-actin expression. Pretreatment of rat MCs with PD98059 (10 μM) compromised the mannitol-inhibited myocardin and SM α-actin protein levels (Figure 6D & 6E), as detected by Western blot. Consistently, 5 μM U0126 also compromised the myocardin and SM α-actin protein levels downregulated by 30 mM D-glucose in rat MCs (data not shown).

## DISCUSSION

**Figure 7:**
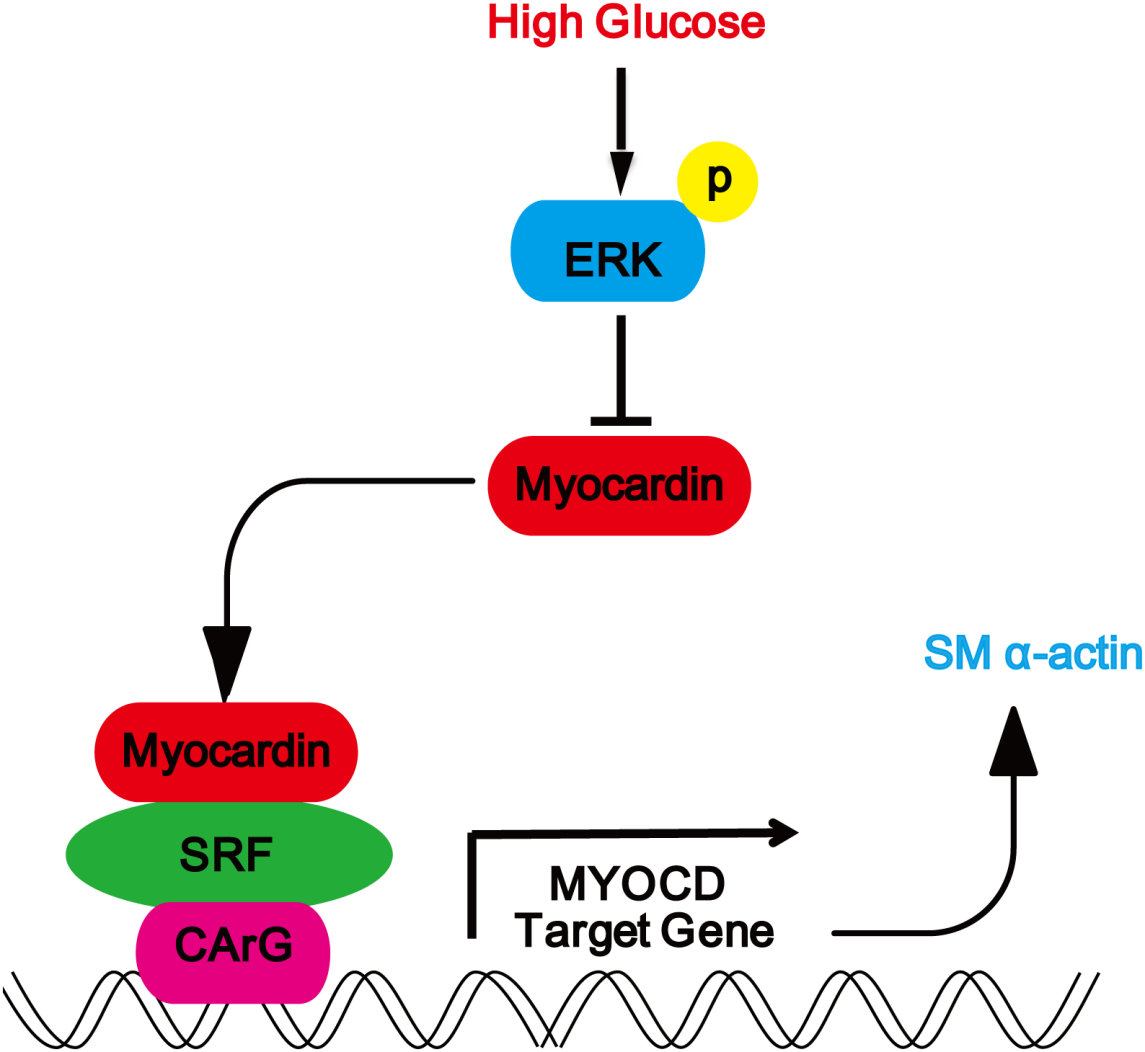
Schematic representation showing that high glucose downregulates the expression myocardin via the ERK signaling pathway in rat glomerular MCs Schematic representation showing the high glucose-induced signaling pathway in rat glomerular MCs. High glucose activates the ERK signaling pathway to downregulate the expression of myocardin and downstream SM α-actin.

To the best of our knowledge, this is the first report showing that *myocardin* is expressed in rat glomerular MCs and that high glucose downregulated the mRNA and protein expression of myocardin and the myocardin target contractile gene SM α-actin through the ERK signaling pathway (Figure 7). Downregulation of myocardin and SM α-actin expression by high glucose in rat MCs suggests a role for myocardin in the pathogenesis of DN.

*Myocardin* is expressed in the heart and in many developing and adult SMC compartments such as the uterus and ladder [15]. Evidence has suggested that myocardin may be expressed in other cells or tissues. For example, thrombin induces myocardin and smooth muscle myosin heavy chain protein expression in peripheral blood mononuclear cells via the RhoA signaling pathway [16]. In the kidney, Saleem et al. reported that podocytes consistently express the differentiated smooth muscle markers smoothelin and calponin and the specific transcription factor myocardin both *in vitro* and *in vivo* [10]. Our results revealed that *myocardin* is also expressed in glomerular mesangial cells. Considering myocardin plays a critical role in SMC contractility and contributes to the pathogenesis of cardiovascular disease, our findings further support a novel mechanism for high glucose in inhibiting MC contraction as well as a role in DN. Because mice without myocardin are viable [17], it will be interesting to examine whether DN develops in these animals.

Previous studies have demonstrated that downregulated contractile proteins, such as SM α-actin and SM22, contribute to restraining the contractility of smooth muscles cells [9, 18]. Our lab also showed that downregulation of myocardin and its target genes, such as SM22, is correlated with contractile inhibition in mouse vascular tissues [9]. Consistently, our results also revealed that high glucose treatment significantly downregulates the expression of myocardin and SM α-actin in rat MCs, strongly indicating that the downregulation of myocardin expression induced by high glucose is related to hypo-contractility and therefore plays a potential role in the pathogenesis of DN.

Mannitol is a type of sugar alcohol that is also used as a medication. Mannitol has a similar osmotic potential as glucose; thus, we had intended to use mannitol as an isotonic control. Unexpectedly, we found that it plays a role similar to that of high glucose in rat MCs. Treatment with 30 mM mannitol and 30 mM D-glucose decreased the expression of myocardin and SM α-actin through activation of the ERK signaling pathway; in contrast, mannitol did not downregulate *myocardin* mRNA levels in rat MCs. Mannitol is clinically used in patients with end-stage renal failure. The therapeutic effect of mannitol may be achieved by reducing glomerular blood viscosity and osmotic potential, as well as increasing urinary flow and preventing the formation of urinary obstructions [19–22]. Our results suggest that mannitol increases the GFR by downregulating the expression of myocardin and the contractile gene SM α-actin, which may provide another potential mechanism for mannitol in therapy for renal disease. Notably, L-glucose, another isotonic control, did not affect *myocardin* and *SM α-actin* mRNA and protein levels, which confirms that the effects of high glucose on myocardin and SM α-actin is not due to the osmotic potential. However, the precise function of osmotic potential is DN needs to be clearly demonstrated in further studies.

The ERK signaling pathway is widely studied and plays a vital role in living cells. It has been reported that SM α-actin is upregulated by arginine vasopressin, angiotensin II and endothelin-1 in 3-dimensional matrix-cultured MCs via the ERK pathway [12]. The inhibition of SM α-actin protein levels induced by PDGF was restored by an ERK inhibitor in primary human bronchial smooth muscle [23]. Moreover, activation of the ERK pathway in hyperglycemia induced both DN and chronic renal failure [14]. Our data revealed that the ERK pathway is activated by high glucose in rat MCs, which contributes to the downregulation of myocardin and SM α-actin expression. Therefore, our results further support a role for myocardin downregulation in DN.

Taken together, our results revealed that *myocardin* is expressed in rat glomerular MCs. High glucose and mannitol downregulated myocardin and SM α-actin expression via the ERK signaling pathway. Given the repressive effects of high glucose on myocardin expression, we speculate that high glucose may result in the downregulation of myocardin *in vivo*, contributing to the reduced MC contractility in DN. Strategies for targeting myocardin in parallel with the production of more specific ERK inhibitors may provide alternative therapeutic approaches for the treatment of DN.

## MATERIALS AND METHODS

### Materials

Glucose, mannitol, antibodies against myocardin, SM α-actin and α-tublin, antibiotics and ERK inhibitor PD98059 were purchased from Sigma-Aldrich (St. Louis, MO). The phospho-p44/42 (ERK) MAPK (Thr202/Tyr204) and total p44/42 (ERK) MAPK antibodies were purchased from Cell Signaling (Beverly, MA). The horseradish peroxidase (HRP)-conjugated secondary antibodies, DMEM low glucose medium and fetal bovine serum (FBS) were from Hyclone Laboratories (Logan, Utah). Reagents for reverse transcription, SYBR Premix, *Ex Taq* and primers were purchased from Qiagen (Valencia, CA). Primers for quantitative PCR and RT-PCR are as following: myocardin, 5′-CAGAAAGTGACAAGAACGATACAG-3′ (forward), 5′-TGAAGCAGCCGAGCATAGG-3′ (reverse) [24], SM α-actin, 5′-CGGGCTT TGCTGGTGATG-3′ (forward), 5′-GGTCAGGATCCCTCTCTTGCT -3′ (reverse) [25], β-actin 5′-CGTGGGCCGCC CTAGGCACCA -3′ (forward), 5′-TTGGCCTTA GGGTTCAGGGGG-3′ (reverse) [26].

### Immunofluorescence analysis

Cells were fixed with 4% paraformaldehyde and permeabilized with 0.5% Triton X-100, followed by blocking with 5% skim milk. Thereafter, the cells were incubated with primary antibody for myocardin (dilution: 1:200) at 37°C for 1 h and fluorescein isothiocyanate (FITC) conjugated secondary antibody (dilution: 1:200). The nuclei were counterstained with 406-diamidino-2-phenylindole (DAPI). Image stacks were acquired by confocal microscopy (Olympus, FV1000).

### Cell culture

Rat glomerular MCs were obtained from China Center for Type Culture Collection (no. HBYZ-1; CCTCC, WuHan, CN). Cells were cultured in DMEM low glucose medium (Hyclone, Logan, UT) with 10 % heat-inactivated FBS (Hyclone, Logan, UT) and 1 % antibiotic solution (Sigma-Aldrich, St. Louis, MO). Cells from passages three to five were used for all the experiments. Monolayer rat glomerular MCs were rendered quiescent in DMEM low glucose medium containing 0 % fetal bovine serum for 24 h. After treated with or without 30 mM D-glucose, mannitol or L-glucose (Sigma-Aldrich, St. Louis, MO), the cells were harvested for the extraction of protein and RNA. The ERK specific inhibitor PD98059 (Sigma-Aldrich, St. Louis, MO) was added to the cells 30 min before treated with 30 mM D-glucose, mannitol or L-glucose. All treatments were performed in DMEM low glucose media containing 0 % FBS. Each experiment was performed in triplicate.

### Reverse transcription (RT) and real-time quantitative polymerase chain reaction (RT-qPCR)

Total RNA in cultured cells was extracted using the RNeasy Mini Kit (QIAGEN, Valencia, CA) according to the manufacturer's instructions. RNA concentrations were determined using a UV spectrophotometer absorbance ratio of 260 to 280 nm (A260/280). RT reactions were carried out using the Reverse Transcriptase System (Promega, Madison, WI) in a 20 μl reaction volume at 42°C for 60 min using a PCR machine (Hangzhou Jingle Scientific Instrument CO. Ltd., Hangzhou, China). All qPCR experiments were performed on a real-time PCR machine (Bio-Rad Laboratories) with the QuantiTect SYBR Green PCR Kit and gene-specific primers purchased from QIAGEN. Quantification of gene expression was assessed by the comparative cycle threshold (Ct) method. The relative amounts of mRNA for the target genes were determined by subtracting the Ct values for these genes from the Ct value for the housekeeping gene β-actin (ΔCt). Data are depicted as 2^−ΔCt^.

### Western blot

Cell cultures were lysed with ice-cold lysis buffer [150 mM NaCl, 50 mM Tris-Cl (pH 7.5), 1 mM EDTA, 1% NP-40, 0.1% SDS, 0.25% sodium deoxycholate, 1 mM PMSF, 1 mM β-glycerophosphate, 1 mM NaF, 1 mM Na_3_VO_4_, and protease inhibitor cocktail tablets (Roche Molecular BioChemicals, Indianapolis, IN)], followed by centrifugation at 4°C for 10 min (12,000 rpm). Protein concentration was determined with a bicinchoninic acid (BCA) Protein Assay Reagent (Pierce, Rockford, IL) according to the manufacturer's instructions. An equal amount of protein from each lysate was mixed with loading buffer, followed by denaturing at 95°C for 5 min. Proteins were separated by 12% sodium dodecyl sulfate polyacrylamide gel electrophoresis (SDS-PAGE), and transferred to polyvinylidene difluoride (PVDF) membranes. The membranes were blocked with 5% nonfat milk in Tris-buffered-Saline/Tween-20 (TBST) buffer (20 mM Tris-HCl, pH 7.6, 136 mM NaCl, and 0.1% Tween-20), and then probed for 1.5 h or overnight with primary antibodies: myocardin: 1:500; SM α-actin: 1:1000; α-tubulin: 1:5,000; phospho-p44/42 (ERK) MAPK: 1:1000; total p44/42 (ERK) MAPK: 1:1000. The membranes were then incubated with horseradish peroxidase-conjugated secondary antibody (dilution: 1:5,000) for 1.5 h at room temperature. Protein signals were visualized using the West Pico Chemiluminescent Substrate Kit (Pierce, Rockford, IL). Images were acquired by a Molecular Image Chemidoc XRS System (Bio-Rad Laboratories) and analyzed using Quantity One soft-ware (Bio-Rad Laboratories).

### Statistical analysis

All experiments were repeated at least three times. The data are presented as means ± SEM. Statistical differences were analyzed using an unpaired Student *t*-test. and *P* value <.05 were considered statistically significant.
